# The Impact of Obesity on the Severity of Clinicopathologic Parameters in Patients with IgA Nephropathy

**DOI:** 10.3390/jcm9092824

**Published:** 2020-08-31

**Authors:** Yu Ah Hong, Ji Won Min, Myung Ah Ha, Eun Sil Koh, Hyung Duk Kim, Tae Hyun Ban, Young Soo Kim, Yong Kyun Kim, Dongryul Kim, Seok Joon Shin, Won Jung Choi, Yoon Kyung Chang, Suk Young Kim, Cheol Whee Park, Young Ok Kim, Chul Woo Yang, Hye Eun Yoon

**Affiliations:** 1 Department of Internal Medicine, Daejeon St. Mary’s Hospital, College of Medicine, The Catholic University of Korea, Daejeon 34943, Korea; amorfati@catholic.ac.kr (Y.A.H.); 1jungchoi@gmail.com (W.J.C.); racer@catholic.ac.kr (Y.K.C.); alterego54@catholic.ac.kr (S.Y.K.); 2 Department of Internal Medicine, Bucheon St. Mary’s Hospital, College of Medicine, The Catholic University of Korea, Bucheon 14647, Korea; blueberi12@gmail.com (J.W.M.); hamyunga@naver.com (M.A.H.); 3 Department of Internal Medicine, Yeouido St. Mary’s Hospital, College of Medicine, The Catholic University of Korea, Seoul 07345, Korea; fiji79@catholic.ac.kr; 4 Department of Internal Medicine, Seoul St. Mary’s Hospital, College of Medicine, The Catholic University of Korea, Seoul 06591, Korea; scamph@catholic.ac.kr (H.D.K.); cheolwhee@hanmail.net (C.W.P.); yangch@catholic.ac.kr (C.W.Y.); 5 Department of Internal Medicine, Eunpyeong St. Mary’s Hospital, College of Medicine, The Catholic University of Korea, Seoul 03476, Korea; deux0123@catholic.ac.kr; 6 Department of Internal Medicine, Uijeongbu St. Mary’s Hospital, College of Medicine, The Catholic University of Korea, Uijeongbu 11765, Korea; dr52916@catholic.ac.kr (Y.S.K.); cmckyo@catholic.ac.kr (Y.O.K.); 7 Department of Internal Medicine, St. Vincent’s Hospital, College of Medicine, The Catholic University of Korea, Suwon 16247, Korea; drkimyk@catholic.ac.kr; 8 Department of Internal Medicine, Incheon St. Mary’s Hospital, College of Medicine, The Catholic University of Korea, Incheon 22711, Korea; imndrkim@gmail.com (D.K.); imkidney@catholic.ac.kr (S.J.S.)

**Keywords:** obesity, mesangial matrix expansion, body mass index, IgA nephropathy

## Abstract

Several studies reported the effect of obesity on the progression of IgA nephropathy (IgAN). However, the impact of obesity on the clinicopathologic presentation of IgAN remains uncertain. This is a retrospective cross-sectional study from eight university hospitals in South Korea. Patients were categorized into three groups using the Asia-Pacific obesity classification based on body mass index (BMI). Clinical and histopathologic data at the time of renal biopsy were analyzed. Among 537 patients with IgAN, the obese group was more hypertensive and had lower estimated glomerular filtration rate and more proteinuria than other groups. The histologic scores for mesangial matrix expansion (MME), interstitial fibrosis, tubular atrophy, and mesangial C3 deposition differed significantly between the three groups. Among these histopathologic parameters, BMI was independently positively associated with MME score on multivariable linear regression analysis (*p* = 0.028). Using multivariable logistic regression analysis, the obese group was independently associated with higher MME scores compared to the normal weight/overweight group (*p* = 0.020). However, BMI was not independently associated with estimated glomerular filtration rate or proteinuria on multivariable analysis. Obesity was independently associated with severe MME in patients with IgAN. Obesity may play an important pathogenetic role in mesangial lesions seen in IgAN.

## 1. Introduction

The prevalence of obesity has been rising regardless of age, sex, and race for the past few decades [1] and is a growing public healthcare concern worldwide [2,3,4]. Obesity has been implicated in the development of several chronic comorbidities, including type 2 diabetes mellitus, hypertension, cardiovascular disease, stroke, dyslipidemia, obstructive sleep apnea, fatty liver and biliary disease, osteoarthritis, and malignancies, leading to increased cardiovascular and all-cause mortality [5,6,7]. Obesity is also associated with a significantly increased risk of chronic kidney disease (CKD) progression and development of end-stage renal disease (ESRD) [8,9].

The renal effects of obesity include both structural and functional adaptations. Excessive body weight is related to intrarenal hemodynamic parameters, namely increased renal blood flow and hyperfiltration. Pathologically, this promotes low glomerular density with glomerulomegaly and thickening of the glomerular basement membrane (GBM) [10,11,12]. These glomerular changes are defined as obesity-related glomerulopathy, a secondary form of focal segmental glomerulosclerosis (FSGS) [13]. Clinically, obesity-related glomerulopathy presents with subnephrotic or nephrotic-range proteinuria without other features of nephrotic syndrome and rapid loss of kidney function [14,15]. In addition to obesity-related glomerulopathy, obesity may be a risk factor for progression of IgA nephropathy (IgAN) [16,17,18,19,20,21]. Most studies demonstrated that excessive BMI, especially a BMI greater than 25 kg/m^2^, was a risk factor for renal disease progression in IgAN patients [16,17]. On the other hand, BMI was not an independent predictor for IgAN progression in some reports [18,22]. Furthermore, recent research showed that underweight, rather than obesity, was an independent risk factor for progression to ESRD, which might be associated with malnutrition status [21]. Therefore, it is difficult to conclude the overall effect of BMI on progression of IgAN with the existing evidence. This may be due to the complex pathophysiological association among obesity, metabolic abnormalities, and renal outcomes.

To date, there are few studies regarding the effect of obesity on histopathological changes in IgAN. Therefore, we conducted a multi-center cohort study using a kidney biopsy registry to elucidate the complex association between obesity and the clinicopathological characteristics of IgAN in Korea. We focused on the impact of obesity on histopathologic and clinical severity at the time of kidney biopsy in IgAN.

## 2. Materials and Methods

### 2.1. Study Design and Data Source

This was a cross-sectional study of a multi-center cohort that included patients older than 18 who underwent kidney biopsy at eight university hospitals affiliated with the College of Medicine Catholic University of Korea between January 2015 and November 2019. This study was conducted in accordance with the Declaration of Helsinki and was approved by the Institutional Review Board of the College of Medicine, Catholic University of Korea (XC19OEDI0025). Written informed consent was obtained from patients at the time of biopsy.

We retrospectively collected records from patients with primary IgAN. We excluded 20 out of 557 patients diagnosed with IgAN whose histologic descriptive form was not in accordance with others. A total of 537 patients were finally enrolled in the present study. Patients with Henoch-Schonlein purpura nephritis were ineligible. Patients were divided into three groups according to body mass index (BMI). BMI was calculated as (weight in kilograms)/(height in meters)^2^. Subjects were categorized according to the Asia-Pacific obesity classification as follows: underweight (<18.5 kg/m^2^), normal weight/overweight (18.5–24.9 kg/m^2^), and obese (≥25 kg/m^2^) [23]. The BMI cut-offs in the World Health Organization (WHO) classification have categorized BMI 25–29.9 as overweight and ≥30 kg/m^2^ as obese [24]. The Asia-Pacific classification of BMI has a lower cut-off for overweight and obese categories compared to the WHO classification. Because the Korean Society for the Study of Obesity defined obesity as BMI ≥ 25 kg/m^2^ according to the Asia-Pacific obesity classification [25], we used a cut-off point for BMI of 25 kg/m^2^ in the present study. A flow diagram for patient selection is presented in Figure S1 (Supplementary Materials).

### 2.2. Data Collection and Definitions

Using the database from the Kidney Biopsy Registry of Catholic Medical Center, all patients were admitted for kidney biopsy and we collected the following data at the time of kidney biopsy. Baseline demographics and clinical data ([including age, sex, height, weight, BMI, co-morbidities, systolic blood pressure (SBP), diastolic blood pressure (DBP), laboratory data, and treatment characteristics) were collected. Blood chemistry data included hemoglobin, high sensitivity-C-reactive protein (hs-CRP), fasting glucose, creatinine, albumin, aspartate aminotransferase (AST), alanine aminotransferase (ALT), uric acid, total cholesterol, triglycerides, low density lipoprotein-cholesterol (LDL-C), high density lipoprotein-cholesterol (HDL-C), serum complement 3 (C3), complement 4 (C4), and serum IgA levels. Proteinuria was assessed by 24 h urine collection. The estimated glomerular filtration rate (eGFR) was calculated from the Chronic Kidney Disease Epidemiology Collaboration (CKD-EPI) equation [26]. The degree of urinary red blood cells (RBC) sediment was scored in five phases, as follows: <3 RBCs/high power field (HPF), 0; 3–5 RBCs/HPF, 1; 5–9 RBCs/HPF, 2; 10–19 RBCs/HPF, 3; and more than 19 RBCs/HPF, 4. We also assessed treatment plans after the diagnosis of IgAN, including use of anti-hypertensive medications, diuretics, lipid-lowering drugs, and immunosuppressive medications.

### 2.3. Histopathologic Parameters

All kidney tissue specimens were obtained by percutaneous needle biopsy. Pathologic slides were reviewed by expert renal pathologists in each center. On light microscopy, the total number of glomeruli and the percentage of glomerulosclerosis and crescents were assessed quantitatively. Renal histologic findings were scored according to the histologic grading system from our university as follows. The severity of mesangial matrix expansion (MME), mesangial cell proliferation, endocapillary proliferation, interstitial fibrosis (IF), tubular atrophy (TA), arterial intimal hyalinosis, and fibrous vessel wall thickening were semi-quantitatively graded from 0 to 4 as follows: grade 0, absent; grade 1, trace (<20%); grade 2, mild (20%–40%); grade 3, moderate (40%–70%); and grade 4, severe (≥70%). Renal biopsy findings were also assessed according to the Oxford classification (MEST score) [27] or the WHO classification (class I to VI) [28]. On immunofluorescence microscopy, the severity of mesangial deposition of IgA, C3, and C4d were graded as 0 (absent), +1 (trace), +2 (mild), +3 (moderate), and +4 (marked).

### 2.4. Statistical Analysis

Continuous data were expressed as mean ± standard deviation and were compared using one-way ANOVA followed by Scheffe’s post-hoc comparisons (parametric) or Kruskal–Wallis test (non-parametric) as appropriate. Categorical data were expressed as numbers (percentage) and compared using the chi-squared test. Spearman correlation coefficients were calculated for correlations between BMI and clinical/laboratory variables. Linear regression analyses were performed to evaluate the association between BMI and histopathologic parameters and the associations between eGFR and 24 h proteinuria and clinicopathological parameters. Logistic regression analyses were performed to estimate the odds ratios (ORs) and 95% confidence intervals (CIs) for high grades of MME, IF, and TA, and positive mesangial deposition of C3 and IgA according to the BMI groups. High-grade MME, IF, and TA were defined as grades 2–4, and positive mesangial C3 and IgA deposition was defined as scores ≥ +2. A *p* value of <0.05 was considered statistically significant. The statistical analyses were performed using SPSS version 20.0 software (SPSS, Inc., Chicago, IL, USA).

## 3. Results

### 3.1. Baseline Characteristics of the Study Subjects Stratified by BMI

The mean age of the subjects was 41.2 ± 14.7 years, and the mean BMI was 24.1 ± 4.1 kg/m^2^. Among 537 patients, 32 patients (5.9%) were underweight, 312 patients (57.8%) were normal weight or overweight, and 193 patients (35.7%) were obese. The baseline clinical characteristics of the study population were compared between the three groups according to BMI classification (Table 1). The obese group was older and had a higher prevalence of hypertension than the underweight and normal weight/overweight groups. SBP and DBP and blood levels of hemoglobin, fasting glucose, liver enzymes, uric acid, total cholesterol, triglycerides, LDL-C, 24-h proteinuria, C3, and C4 were higher in the obese group compared to the other groups, while the eGFR was lower. Figure S2 shows the distribution of BMI according to age and sex categories. For all patients, the BMI distribution peak was shifted to the left. Patients with BMI ≥ 25 kg/m^2^ accounted for 35.9% of the total subjects (40.4% in males and 31.8% in females). Patients who had lower BMI tended to be younger, and the distribution of BMI was similar between males and females.

### 3.2. Histopathologic Findings of the Study Subjects Stratified by BMI

**Figure 1:**
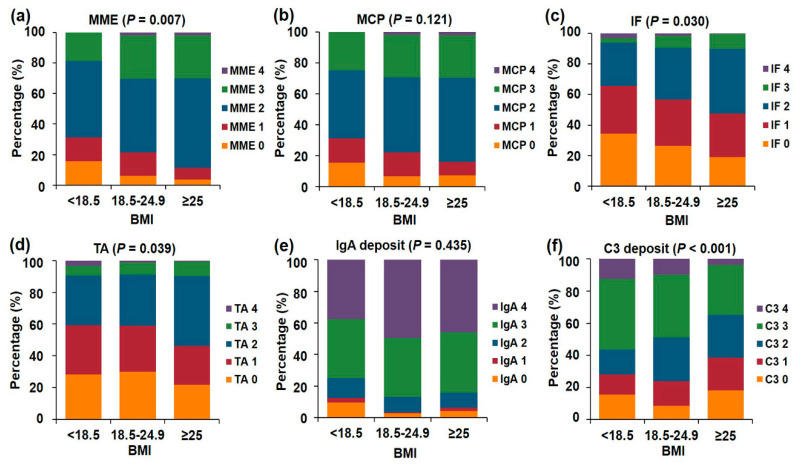
The distribution of histopathologic scores according to the three BMI groups. (**a**) Mesangial matrix expansion (MME). (**b**) Mesangial cell proliferation (MCP). (**c**) Interstitial fibrosis (IF). (**d**) Tubular atrophy (TA). (**e**) IgA mesangial deposition. (**f**) C3 mesangial deposition.

We compared histopathologic features between the three BMI groups in Table 2 and Table 3 and Figure 1. Total number of glomeruli and the mean mesangial C3 deposition score were lower (*p* = 0.003 and *p* < 0.001, respectively), and the mean MME (*p* = 0.042), IF (*p* = 0.046) and TA (*p* = 0.033) score was higher in the obese group compared to the other groups (Table 2). On light microscopy, the glomeruli, mesangium, tubules, interstitium, vessels, and mesangial IgA deposition score did not differ between the three groups. There was an increasing trend of high grade MME (*p* = 0.007), IF (*p* = 0.03), and TA (*p* = 0.039) as the BMI increased. The distribution of the C3 deposition severity was different among the three groups (*p* < 0.001, Figure 1). The distributions for WHO and Oxford classifications of IgAN were not significantly different between the three BMI groups (Table 3).

### 3.3. Association between BMI and Histopathologic Parameters

Univariable linear regression analysis showed that scores for MME, endocapillary proliferation, IF, and TA were positively correlated with BMI, while the number of total glomeruli and the mesangial C3 deposition score were negatively correlated with BMI (Table 4). Multivariable linear regression analysis showed that the MME and mesangial IgA deposition scores were positively associated with BMI (*p* = 0.028; adjusted R^2^ = 0.291 and *p* = 0.041; adjusted R^2^ = 0.291, respectively), while total number of glomeruli was negatively associated with BMI (*p* = 0.029; adjusted R^2^ = 0.286) after adjusting for clinical parameters including age, SBP, hemoglobin, glucose, albumin, AST, ALT, uric acid, total cholesterol, eGFR, 24-h proteinuria, and serum C3, C4, and IgA levels.

Table 5 shows the OR and 95% CI for high MME, IF, TA, and positive mesangial deposition of C3 and IgA for the three BMI groups. Considering the normal weight or overweight group as a reference, the obese group showed higher risks for high MME and TA, and lower risk for mesangial C3 deposition. After adjusting for age, sex, the presence of hypertension and diabetes mellitus, and SBP (Model 1), the obese group exhibited higher risk for severe MME (OR = 2.066, 95% CI 1.227–3.478, *p* = 0.006) and lower risk for mesangial C3 deposition (OR = 0.544, 95% CI 0.365–0.810, *p* = 0.003). After adjusting for model 1 with total glomerular number, eGFR, 24-h proteinuria, and blood levels of hemoglobin, glucose, albumin, AST, ALT, uric acid, total cholesterol, C3, C4, and IgA (Model 2), the obese group still had a significantly higher risk for severe MME (OR = 2.060, 95% CI 1.120–3.788, *p* = 0.020), while the underweight group had a significantly lower risk for severe MME (OR = 0.369, 95% CI 0.150–0.904, *p* = 0.029) and mesangial IgA deposition (OR = 0.208, 95% CI 0.049–0.889, *p* = 0.034).

To evaluate whether the association between BMI and MME in IgAN is an incidental finding in obese patients, we analyzed subjects with tubulointerstitial disease without glomerular injury (acute tubular necrosis, interstitial nephritis, and pyelonephritis) during the same period in our kidney biopsy cohort (n = 80). There was no significant association between BMI groups and MME in subjects with tubulointerstitial diseases (Table S1 (Supplementary Materials)).

### 3.4. Association of BMI with Clinical Variables, Renal Function, and Proteinuria

**Figure 2:**
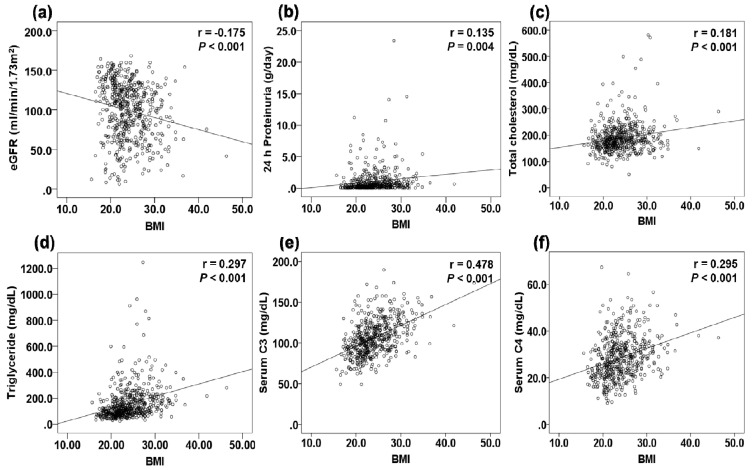
The correlation graphs between BMI and clinical variables. (**a**) CKD-EPI eGFR. (**b**) 24 h proteinuria. (**c**) Total cholesterol level. (**d**) Triglyceride level. (**e**) Serum C3 level. (**f**) Serum C4 level.

There was a positive relationship between BMI and 24 h proteinuria, total cholesterol, triglycerides, and serum C3 and C4 levels and a negative relationship between BMI and eGFR (Figure 2). The association of BMI with renal function and proteinuria was evaluated by adjusting for clinical and histopathological variables in linear regression analysis (Table 6). BMI was negatively correlated with eGFR in univariable analysis; however, this relationship was not significant on multivariable analysis. Factors significantly associated with eGFR were age, SBP, levels of serum albumin and HDL-C, total number of glomeruli, and the interstitial fibrosis score (adjusted R^2^ = 0.460). BMI showed a positive linear association with 24-h proteinuria on univariable analysis, but the association was not significant on multivariable analysis. eGFR and serum levels of albumin and C3 were significantly associated with 24-h proteinuria (adjusted R^2^ = 0.260).

### 3.5. Treatment Patterns of the Study Subjects Stratified by BMI

At the time of kidney biopsy, there was no significant difference in the use of renin-angiotensin-aldosterone system (RAAS) blockers among the three groups (*p* = 0.252); BMI < 18.5 kg/m^2^ (n = 13, 40.6%) vs. 18.5–24.9 kg/m^2^ (n = 87, 27.9%) vs. ≥ 25 kg/m^2^ (n = 51, 26.4%). Several treatment strategies were chosen within 6 months after kidney biopsy and these were compared between the three groups. More patients in the obese group were treated with RAAS blockers, calcium channel blockers (CCBs), β-blockers, thiazide, statins, and corticosteroids compared to the underweight and normal/overweight groups (Table 7).

## 4. Discussion

In this study, we evaluated the histopathological and clinical implications of obesity in patients with IgAN. Intriguingly, multivariable logistic regression demonstrated that a BMI greater than 25 kg/m^2^ was independently associated with high MME in patients with IgAN. Patients with elevated BMI were more hypertensive and had increased proteinuria, but the association of BMI with renal function and proteinuria was modest in IgAN.

Only a few studies have investigated the influence of obesity on histological parameters in IgAN. First, Bonnet et al. suggested that vascular, tubular, and interstitial indices were higher in obese patients than non-obese patients using a semi-quantitative classification scheme, but the glomerular index did not differ significantly. A recent cohort analysis of 481 IgAN patients also showed that high BMI was associated with a high risk of IF [22]. These previous studies did not focus and evaluate the association between increased BMI and MME in IgAN. Tanaka et al. [29] also reported that obese patients with IgAN showed significantly increased proteinuria, accompanied by GBM thickening and glomerulomegaly, mimicking obesity-related glomerulopathy. This study showed no significant differences in the severity of mesangial proliferation and matrix expansion between obese and non-obese group, but the number of enrolled patients were very small. Our study is the first study to show an independent association between obesity and MME in IgAN. Moreover, in subjects with tubulointerstitial disease, obesity did not show a significant association with MME, which means that the association between obesity and MME in IgAN is not an incidental finding seen in obese patients. Although the precise mechanism by which obesity causes MME remains unclear, obesity can lead to a rise in the concentration of leptin, which stimulates the expression of transforming growth factor-β1 (TGF-β1). TGF-β1 is known to act as the main driver of extracellular matrix accumulation, mesangial cell proliferation, and progressive glomerulosclerosis [30,31]. These in turn may be responsible for the proliferation of mesangial cells and expanded mesangial matrix. MME has been regarded as a step towards glomerular sclerosis [32]. Although FSGS has long been recognized as the hallmark lesion of obesity-related glomerulopathy [13], Serra et al. [33] also found that increased mesangial matrix, podocyte hypertrophy, and mesangial cell proliferation were more frequent in extremely obese patients compared to normal weight controls. Of note, our study supports these findings in that obesity could independently increase MME after adjustment for confounding factors and strengthens our knowledge of the pathogenic role of obesity in mesangial lesions in IgAN.

Obesity-induced subclinical inflammation is responsible for activation of the complement system [34]. C3 and C4 are expressed and secreted by adipose tissue [35], and serum C3 is strongly associated with components of metabolic syndrome, such as insulin resistance, lipid profile, and BMI [36,37,38]. Furthermore, weight gain increases C3, which decreases upon weight loss [39]. Therefore, C3 has been linked to the etiology of obesity, and to a wide range of obesity sequelae. In this study, serum C3 and C4 levels were increased in patients with higher BMI. Serum C3 level was positively associated with 24 h proteinuria using multivariable analysis. Consistent with our study, some researchers previously reported that serum C3 and C4 levels were positively correlated with increased BMI and severe proteinuria in IgAN patients [19,21]. High serum C4 level was also associated with severe proteinuria and renal pathological damage in IgAN [40]. Altogether, these findings suggest that obesity may activate the complement system and is related to the severity of renal presentation in patients with IgAN.

In this study, mesangial C3 deposition decreased as BMI increased, but multivariable linear regression and logistic regression analyses showed that mesangial C3 deposition was not independently associated with BMI. Additionally, mesangial C3 deposition was not significantly associated with eGFR or 24 h proteinuria. Therefore, we speculate that the effect of obesity on the activation of the local complement system is modest in IgAN. Previous studies revealed conflicting results, suggesting an important role of local complement activation in the pathogenesis of IgAN. Mesangial C3c deposition was associated with active inflammation in IgAN [41], and mesangial C3 deposition was an independent risk factor for progression of IgAN [42]. A recent study also reported that mesangial C3 deposition was associated with poor outcome in IgAN; however, eGFR or proteinuria at the time of kidney biopsy did not differ according to the degree of mesangial C3 deposition [43]. The implications of mesangial complement deposition may have differential effects on renal presentation and prognosis, and further analysis is needed to clarify the pathologic role of mesangial complement deposition in IgAN.

Another interesting finding is that obesity was not independently associated with clinical parameters such as renal function and proteinuria in IgAN. In this study, obese patients showed decreased eGFR and increased 24 h proteinuria compared to other groups, but there were no significant relationships between BMI and eGFR or 24-h proteinuria in multivariable analysis. In addition, the histologic classification of IgAN, such as the Oxford classification or WHO classification, was not different between the groups. Previous studies demonstrated similar findings to our results. A prospective cohort study of 331 French IgAN patients showed that patients with elevated BMI had worse renal presentations and outcomes, but that obesity, per se, did not directly affect these outcomes [18]. In a study of 481 Chinese IgAN patients, there was no significant difference in clinical parameters or Oxford classification scores among BMI groups [22]. In a study including a small number of IgAN patients, obesity had no significant impact on serum creatinine levels or pathological severity in multiple linear regression models [44]. These findings suggest that obesity may not be independently related to decreased renal function, increased proteinuria, or severe forms of histologic classification in IgAN, although it likely affects histologic damage. Obese patients may simultaneously have multiple unhealthy lifestyle behaviors, such as smoking, increased energy intake, low physical activity, and various medical conditions including hypertension, diabetes mellitus, and dyslipidemia, which are known risk factors for renal disease progression [45]. In this study, we also showed that obese patients with IgAN had more the presence of hypertension and dyslipidemia, and were more actively treated with supportive managements, such as anti-hypertensive medications, thiazide, and statins, as well as corticosteroids, than underweight or normal/overweight patients. Therefore, increased weight alone may not be enough to induce severe renal parameters and histologic classifications. To elucidate a direct association between obesity and long-term kidney damage, we need to perform further studies involving a larger number of patients with IgAN.

This study has several limitations. First, our findings are limited by the cross-sectional and retrospective design, uncertain causality, and possible influence of confounding factors that could not be captured; in addition, there were no longitudinal follow-up data. Therefore, a prospective, multi-center, large scale study with a longer follow-up period is required to define the histopathological impact of BMI more clearly in IgAN. Second, although this study included eight university hospitals, the evaluation of renal biopsy specimens was conducted by expert renal pathologists in each center. Therefore, there may be interobserver variability. However, each hospital shares the same renal biopsy report form, and the pathologists at each hospital were trained by one pathologist. Thus, observer bias in histologic assessment may be relatively small. To compensate for this limitation, the histologic grading and staging of IgAN were described according to both the Oxford and WHO classifications. Third, the inclusion of only Korean patients may limit the generalizability of our findings to other ethnic populations. Current practice guidelines suggested that obesity is typically defined quite simply as excess body weight for height, but this simple definition of obesity cannot reflect the consideration of age or race/ethnicity. Therefore, further studies may be needed to elucidate the association with obesity and clinicopathologic parameters in various ethnic groups of IgAN. Fourth, we did not measure the anthropometric parameters associated with metabolic syndrome, such as waist circumference, skin fold thickness and waist-to-hip ratio, and the index of insulin resistance in this study. Further studies will be required to determine whether these markers may be more reliable measures than BMI in IgAN.

## 5. Conclusions

In conclusion, our study showed that obesity was independently associated with MME in Korean IgAN patients and these findings suggest that obesity may have an important pathogenetic role in mesangial lesions in IgAN.

## Figures and Tables

**Table 1:** Baseline clinical variables of the three BMI groups at the time of renal biopsy.

	BMI			
	<18.5 kg/m^2^ (n = 32)	18.5–24.9 kg/m^2^ (n = 312)	≥25 kg/m^2^ (n = 193)	*p*
Age (year)	34.3 ± 14.8	40.0 ± 15.2 *	44.1 ± 13.3 *	<0.001
Sex (male, %)	15 (46.9)	140 (44.9)	105 (54.4)	0.112
Alcohol (yes, %)	7 (21.9)	56 (17.9)	39 (20.3)	0.737
Smoking (yes, %)	7 (21.9)	42 (13.5)	35 (18.7)	0.219
Diabetes mellitus (%)	3 (9.4)	14 (4.5)	15 (7.8)	0.218
Hypertension (%)	5 (15.6)	75 (24.0)	74 (38.5)	0.001
BMI (kg/m^2^)	17.7 ± 0.7	22.0 ± 1.7 *	28.5 ± 3.1 *^,†^	<0.001
SBP (mmHg)	115.5 ± 15.5	122.9 ± 16.5 *	127.8 ± 14.5 *	<0.001
DBP (mmHg)	69.8 ± 11.6	75.3 ± 10.0 *	78.8 ± 10.0 *	<0.001
Hemoglobin (g/dL)	12.7 ± 1.9	13.0 ± 2.0 *	13.8 ± 1.8 *	<0.001
hs-CRP (mg/dL)	0.6 ± 0.6	0.7 ± 4.3	0.8 ± 2.4	0.783
Glucose (mg/dL)	96.7 ± 14.5	105.1 ± 29.5	113.3 ± 37.2 *	0.004
Serum creatinine (mg/dL)	1.1 ± 0.9	1.1 ± 1.0	1.1 ± 0.6	0.945
eGFR (mL/min/1.73 m^2^)	111.1 ± 38.1	101.7 ± 36.9	93.3 ± 32.1*	0.006
Serum albumin (g/dL)	4.1 ± 0.5	4.0 ± 0.6	3.9 ± 0.6	0.303
AST (IU/L)	19.6 ± 5.1	22.3 ± 10.9	24.3 ± 10.0 *	0.021
ALT (IU/L)	12.7 ± 6.2	20.2 ± 28.0	25.9 ± 19.6 *	0.003
Serum uric acid (mg/dL)	5.3 ± 1.9	5.9 ± 1.9	6.5 ± 2.0 *	<0.001
Total cholesterol (mg/dL)	169.8 ± 38.5	183.7 ± 48.8	201.0 ± 68.0 *	0.001
Triglyceride (mg/dL)	95.5 ± 49.2	132.8 ± 99.5	209.0 ± 163.3 *^,†^	<0.001
LDL-C (mg/dL)	94.4 ± 132.8	104.9 ± 37.1	118.0 ± 46.3 *	<0.001
HDL-C (mg/dL)	59.3 ± 17.0	54.1 ± 16.1	48.4 ± 16.9 *	<0.001
24-h proteinuria (g/day)	0.45 ± 0.85	0.93 ±1.52	1.46 ±2.71 *	0.007
Urine RBCs (grade)	3.0 ± 1.5	2.8 ± 1.6	2.8 ± 1.6	0.697
Serum C3 (mg/dL)	87.2 ± 17.1	101.1 ± 19.6 *	117.8 ± 20.7 *^,†^	<0.001
Serum C4 (mg/dL)	22.7 ± 5.4	27.4 ± 9.0 *	31.6 ± 8.9 *^,†^	<0.001
Serum IgA (mg/dL)	284.0 ± 109.0	316.1 ± 164.3	310.7 ± 147.6	0.475

* ALT, alanine aminotransferase; AST, aspartate aminotransferase; BMI, body mass index; C3, complement 3; C4, complement 4; DBP, diastolic blood pressure; eGFR, estimated glomerular filtration rate; HDL-C, high density lipoprotein-cholesterol; hs-CRP, high sensitivity-C-reactive protein; IgA, immunoglobulin A LDL-C, low density lipoprotein-cholesterol; RBCs, red blood cells; SBP, systolic blood pressure. * *p* < 0.05 vs. BMI < 18.5 kg/m^2^ and ^†^ *p* < 0.05 vs. BMI 18.5–24.9 kg/m^2^ by one-way ANOVA with Scheffe’s post-hoc analysis.

**Table 2:** Histopathological findings of the three BMI groups.

	BMI			
	<18.5 kg/m^2^	18.5–24.9 kg/m^2^	≥25 kg/m^2^	*p*
Total glomerular number	15.9 ± 8.9	13.9 ± 7.9	12.1 ± 7.1 *^,^^†^	0.003
Light microscopy				
Glomerulosclerosis, Total (%)	20.1 ± 27.8	24.3 ± 24.6	26.9 ± 25.1 *	0.101
Global sclerosis (%)	10.2 ± 20.4	16.7 ± 19.4*	17.8 ± 19.9 *	0.015
Segmental sclerosis (%)	7.3 ± 9.7	8.3 ± 13.0	9.5 ± 14.5	0.731
Mesangial matrix expansion (0–4)	1.7 ± 1.0	2.1 ± 0.9	2.2 ± 0.7 *	0.042
Mesangial cell proliferation (0–4)	1.8 ± 1.1	2.0 ± 0.9	2.1 ± 0.9	0.292
Crescents (%)	1.6 ± 4.7	2.6 ± 8.0	2.0 ± 7.5	0.951
Interstitial fibrosis (0–4)	1.1 ± 1.0	1.3 ± 1.0	1.4 ± 0.9 *	0.046
Tubular atrophy (0–4)	1.3 ± 1.0	1.2 ± 1.0	1.4 ± 0.9 ^†^	0.033
Arterial intimal hyalinosis (0–4)	0.3 ± 0.6	0.2 ± 0.6	0.3 ± 0.8	0.213
Fibrous wall thickening (0–4)	0.3 ± 0.7	0.5 ± 0.9	0.6 ± 1.0	0.269
Immunofluorescence microscopy				
IgA Mesangial deposit (0–4)	2.9 ± 1.2	3.3 ± 0.9	3.2 ± 1.0	0.177
C3 Mesangial deposit (0–4)	2.3 ± 1.3	2.3 ± 1.1	1.8 ± 1.2 *^,^^†^	<0.001
C4d Mesangial deposit (0–4)	0.1 ± 0.2	0.2 ± 0.1	0.1 ± 0.4	0.903

* BMI, body mass index; C3, complement 3; C4, complement 4; IgA, immunoglobulin A. * *p* < 0.05 vs. BMI < 18.5 kg/m^2^ and ^†^ *p* < 0.05 vs. BMI 18.5–24.9 kg/m^2^ by post-hoc analysis of Kruskal-Wallis test.

**Table 3:** The distribution of histological classifications according to the three BMI groups.

	BMI			
	<18.5 kg/m^2^	18.5–24.9 kg/m^2^	≥25 kg/m^2^	*p*
WHO classification (n = 436)	n = 22	n = 255	n = 159	
Grade (1–6)	2.91 ± 0.92	2.93 ± 0.93	2.98 ± 0.86	0.834
Oxford classification (n = 173)	n = 10	n = 95	n = 68	
M0 (%)	4 (40)	51 (53.7)	33 (48.5)	0.630
M1 (%)	6 (60)	44 (46.3)	35 (51.5)	
E0 (%)	6 (60)	73 (76.8)	50 (73.5)	0.492
E1 (%)	4 (40)	22 (23.2)	18 (26.5)	
S0 (%)	3 (30.0)	53 (55.8)	29 (42.6)	0.117
S1 (%)	7 (70.0)	42 (44.2)	39 (57.4)	
T0 (%)	7 (70.0)	72 (75.8)	50 (73.5)	0.778
T1 (%)	3 (30.0)	18 (18.9)	16 (23.5)	
T2 (%)	0 (0)	5 (5.3)	2 (2.9)	

BMI, body mass index; WHO, World Health Organization. Oxford classification: M; mesangial hypercellularity, E; endocapillary proliferation, S; segmental sclerosis, T tubular atrophy/interstitial fibrosis. Statistical analysis was performed by Kruskal-Wallis test in WHO classifications and using the chi-squared test in Oxford classifications.

**Table 4:** Linear regression analysis for BMI and the histopathologic parameters.

	BMI					
	Univariable			Multivariable		
	β	t	*p*	β	t	*p*
Total glomerular number	−0.162	−3.802	<0.001	−0.092	−2.192	0.029
Mesangial matrix expansion	0.086	2.004	0.046	0.091	2.205	0.028
Mesangial cell proliferation	0.048	1.112	0.266	0.066	1.585	0.114
Segmental sclerosis	0.040	0.934	0.351	0.062	1.428	0.154
Endocapillary proliferation	0.128	2.974	0.003	0.052	1.236	0.217
Interstitial fibrosis	0.087	2.020	0.044	0.028	0.547	0.585
Tubular atrophy	0.099	2.286	0.023	0.036	0.701	0.484
IgA Mesangial Deposit	−0.025	−0.5825	0.561	0.086	2.048	0.041
C3 Mesangial Deposit	−0.151	−3.521	<0.001	0.008	0.187	0.852
C4d Mesangial Deposit	0.026	0.588	0.557	0.004	0.086	0.932

BMI, body mass index, C3, complement 3; C4d, cleavage product of complement 4; IgA, immunoglobulin A. Multivariable analysis was adjusted for each histologic parameter and clinical parameters, including age, systolic blood pressure, hemoglobin, glucose, albumin, AST, ALT, uric acid, total cholesterol, eGFR, 24-h proteinuria, and serum C3, C4 and IgA levels.

**Table 5:** Logistic regression analysis for BMI groups and histopathologic parameters.

	Crude OR		Model 1		Model 2	
	OR (95% CI)	*p*	OR (95% CI)	*p*	OR (95% CI)	*p*
Mesangial matrix expansion						
<18.5	0.595 (0.269–1.318)	0.201	0.586 (0.259–1.327)	0.200	0.369 (0.150–0.904)	0.029
18.5–24.9	1 (Ref.)		1 (Ref.)		1 (Ref.)	
≥25	2.201 (1.249–3.539)	0.005	2.066 (1.227–3.478)	0.006	2.060 (1.120–3.788)	0.020
Interstitial fibrosis						
<18.5	0.685 (0.319–1.471)	0.332	1.084 (0.475–2.472)	0.848	0.568 (0.157–2.056)	0.389
18.5–24.9	1 (Ref.)		1 (Ref.)		1 (Ref.)	
≥25	1.436 (1.000–2.062)	0.05	1.121 (0.761–1.651)	0.564	1.297 (0.759–2.216)	0.341
Tubular Atrophy						
<18.5	0.983 (0.468–2.063)	0.964	1.550 (0.282–1.550)	0.282	0.861 (0.252–2.937)	0.811
18.5–24.9	1 (Ref.)		1 (Ref.)		1 (Ref.)	
≥25	1.679 (1.168–2.413)	0.005	1.338 (0.909–1.969)	0.140	1.644 (0.961–2.812)	0.070
IgA mesangial deposit						
<18.5	0.236 (0.069–0.800)	0.021	0.236 (0.069–0.800)	0.021	0.208 (0.049–0.889)	0.034
18.5–24.9	1 (Ref.)		1 (Ref.)		1 (Ref.)	
≥25	0.508 (0.215–1.199)	0.508	0.553 (0.228–1.344)	0.191	0.871 (0.279–2.714)	0.812
C3 mesangial deposit						
<18.5	0.812 (0.360–1.831)	0.615	0.533 (0.321–1.800)	0.533	0.516 (0.167–1.599)	0.252
18.5–24.9	1 (Ref.)		1 (Ref.)		1 (Ref.)	
≥25	0.506 (0.343–0.749)	<0.001	0.544 (0.365–0.810)	0.003	0.805 (0.469–1.380)	0.430

BMI, body mass index; C3, complement; IgA, immunoglobulin A. Model 1 was adjusted for age, sex, the presence of hypertension, the presence of diabetes mellitus and systolic blood pressure. Model 2 was adjusted for model 1 + total glomerular number, eGFR, 24-h proteinuria, and blood levels of hemoglobin, glucose, albumin, aspartate aminotransferase, alanine aminotransferase, uric acid, total cholesterol, and serum C3, C4, and serum IgA levels.

**Table 6:** Linear regression analysis for renal function and 24-h proteinuria.

	eGFR						24-h Proteinuria					
	Univariable			Multivariable			Univariable			Multivariable		
	β	t	*p*	β	t	*p*	β	t	*p*	β	t	*p*
Age	−0.438	−11.270	<0.001	−0.273	−7.079	<0.001	0.106	2.283	0.023	-	-	-
SBP	−0.264	−6.315	<0.001	−0.101	−0.265	0.008	0.167	3.629	<0.001	-	-	-
DBP	−0.221	5.224	<0.001				0.088	1.878	0.061	-	-	-
BMI	−0.175	−4.102	<0.001				0.135	2.921	0.004	-	-	-
Hemoglobin	0.306	7.403	<0.001				−0.015	−0.315	0.753	-	-	-
Glucose	−0.089	−2.036	0.042				−0.020	−0.414	0.679	-	-	-
eGFR	-	-	-				−0.272	−6.049	<0.001	−0.120	−2.653	0.008
Albumin	0.413	10.482	<0.001	0.201	5.260	<0.001	−0.455	−10.933	<0.001	−0.429	−9.454	<0.001
Total cholesterol	−0.59	−1.351	0.177				0.193	4.206	<0.001	-	-	-
Triglyceride	−0.125	−2.884	0.004				0.058	1.233	0.218	-	-	-
LDL-C	−0.083	−1.878	0.061				0.216	4.661	<0.001	-	-	-
HDL-C	0.197	4.500	<0.001	0.087	2.314	0.021	0.006	0.122	0.903	-	-	-
Serum C3	0.046	1.035	0.301				0.159	3.390	0.001	0.162	3.851	<0.001
Serum C4	−0.233	−5.389	<0.001				0.170	3.631	<0.001			
Serum IgA	−0.142	−3.166	0.002				−0.017	−0.353	0.724	-	-	-
24-h proteinuria	−0.272	−6.049	<0.001				-	-	-	-	-	-
Total glomerular number	0.103	2.392	0.017	0.109	2.967	0.003	−0.089	−1.909	0.057	-	-	-
Mesangial matrix expansion	−0.065	−1.495	0.136				0.058	1.246	0.213	-	-	-
Mesangial cell proliferation	−0.023	−0.532	0.595				0.059	1.263	0.207	-	-	-
Endocapillary proliferation	−0.108	−2.496	0.013				0.028	0.601	0.548	-	-	-
Interstitial fibrosis	−0.511	−13.678	<0.001	−0.408	−10.284	<0.001	0.191	4.140	<0.001	-	-	-
Tubular atrophy	−0.485	−12.759	<0.001	-	-	-	0.192	4.173	<0.001	-	-	-
Mesangial deposit, IgA	0.104	2.414	0.016	-	-	-	0.012	0.250	0.803	-	-	-
Mesangial deposit, C3	−0.002	−0.046	0.963	-	-	-	0.007	0.142	0.888	-	-	-

BMI, body mass index; C3, complement 3; DBP, diastolic blood pressure; eGFR, estimated glomerular filtration rate; HDL-C, high density lipoprotein-cholesterol; IgA, immunoglobulin A; LDL-C, low density lipoprotein-cholesterol; SBP, systolic blood pressure. The variables included in the multivariable linear regression model were statistically significant variables in the univariable linear regression (*p* < 0.1). Dashes indicate that the variable did not enter the multivariable linear regression model.

**Table 7:** Distribution of treatment after renal biopsy according to the three BMI groups.

	BMI			
	<18.5 kg/m^2^	18.5–24.9 kg/m^2^	≥25 kg/m^2^	*p*
Anti-hypertensive medications				
RAAS blocker	21 (65.6)	223 (71.5)	157 (81.3)	0.029
CCB	3 (9.4)	41 (13.1)	57 (29.5)	<0.001
B-blocker	1 (3.1)	14 (4.5)	22 (11.4)	0.015
Diuretics				
Thiazide	0 (0.0)	1 (0.3)	7 (3.6)	0.033
Furosemide	2 (6.2)	23 (7.4)	21 (10.9)	0.523
Lipid lowering agents				
Statin	6 (18.8)	74 (23.7)	83 (43.0)	<0.001
Omega-3 fatty acid	3 (9.4)	53 (17.0)	38 (19.7)	0.266
Immunosuppressive agents				
Any immunosuppression	24 (75.0)	210 (67.3)	128 (66.3)	0.248
Steroid	4 (12.5)	84 (26.9)	63 (32.6)	0.036

BMI, body mass index; CCB, calcium channer blocker; RAAS, renin-angiotensin-aldosterone system.

## Supplementary Materials

The following are available online at https://www.mdpi.com/2077-0383/9/9/2824/s1, Figure S1: Flow chart of study design. Figure S2: Distribution of BMI according to age and sex categories. Bars of the BMI were divided by age (a) and sex categories (b). Table S1: Logistic regression analysis for BMI groups and MME in tubulointerstitial diseases from our kidney biopsy registry cohort (n = 80).
