# Supplementary material for: The Impact of Obesity on the Severity of Clinicopathologic Parameters in Patients with IgA Nephropathy
Source: J Clin Med. 2020 Aug 31;9(9):2824. doi: 10.3390/jcm9092824 (PMC7564413; doi:10.3390/jcm9092824)
Supplement: Supplementary file 1 [file jcm-09-02824-s001.pdf]

Supplement data

The Impact of Obesity on the Severity of Clinicopathologic Parameters in Patients with IgA Nephropathy

Yu Ah Hong, Ji Won Min, Myung Ah Ha, Eun Sil Koh, Hyung Duk Kim, Tae Hyun Ban, Young Soo Kim, Yong Kyun Kim, Dongryul Kim, Seok Joon Shin, Won Jung Choi, Yoon Kyung Chang, Suk Young Kim, Cheol Whee Park, Young Ok Kim, Chul Woo Yang, and Hye Eun Yoon*

Figure S1. Flow chart of study design.

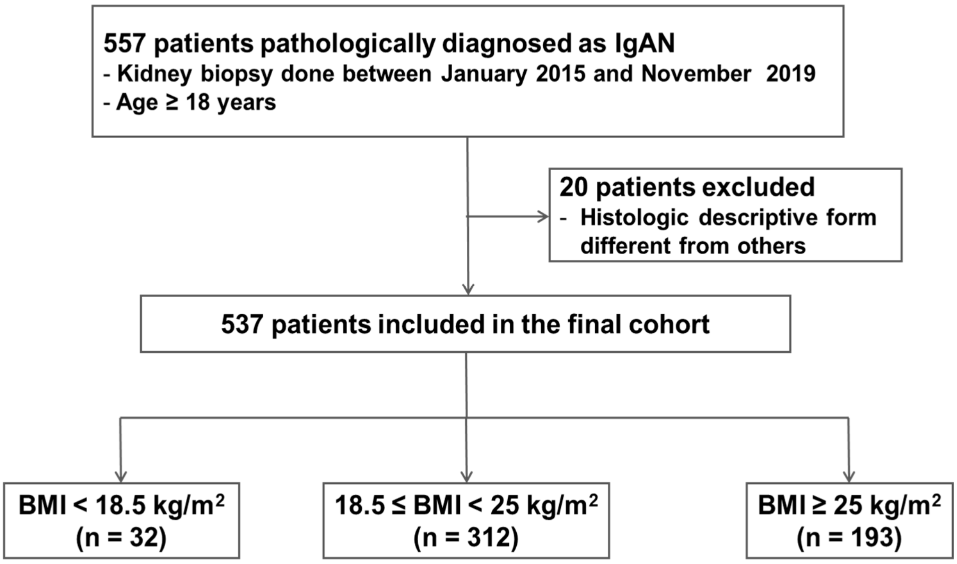

Figure S2. Distribution of BMI according to age and sex categories.

Bars of the BMI were divided by age (a) and sex categories (b).

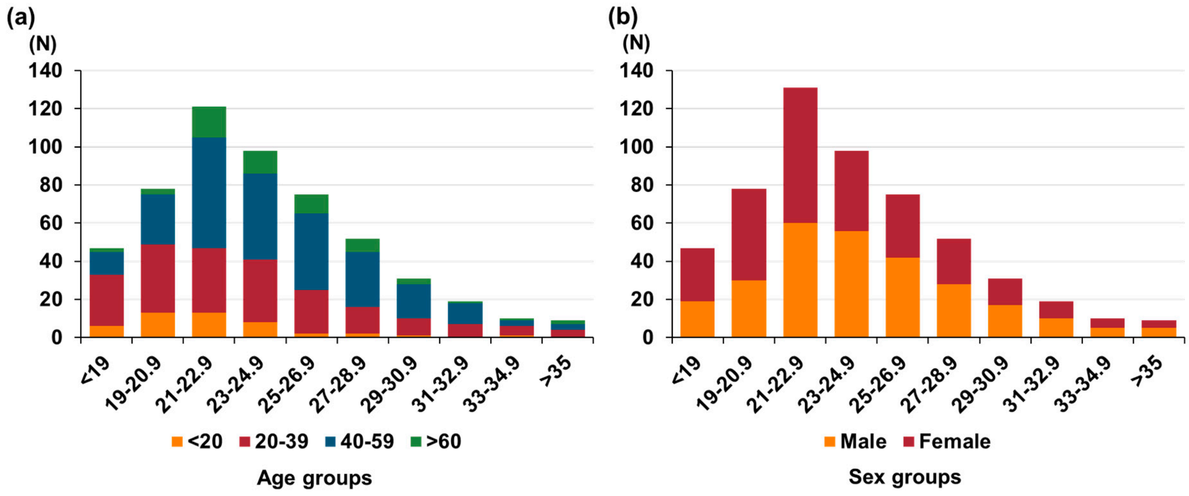

Table S1. Logistic regression analysis for BMI groups and MME in tubulointerstitial diseases from our kidney biopsy registry cohort (n = 80).

	Mesangial Matrix Expansion	
	**Crude OR (95% CI)**	***P***
**BMI < 18.5 kg/m^2**	1.167 (0.096-14.126)	0.904
**BMI 18.5-24.9 kg/m^2**	1 (Ref.)	
**BMI ≥ 25 kg/m^2**	0.875 (0.31.5-2.431)	0.798

* Between January 2015 and November 2019, the number of patients diagnosed as pure tubulointerstitial diseases, such as acute tubular necrosis, acute or chronic interstitial nephritis, and pyelonephritis, was 80 in our cohort.
